# Cholesterol and triglyceride concentrations following 12–18 months of clinically prescribed elexacaftor-tezacaftor-ivacaftor—PROMISE sub-study

**DOI:** 10.1016/j.jcte.2025.100391

**Published:** 2025-04-02

**Authors:** Rosara Bass, Michael Stalvey, George Solomon, Steven Rowe, David Nichols, Sarah Jane Schwarzenberg, Steven Freedman, Rachel Walega, Andrea Kelly

**Affiliations:** a Ohio State University and Nationwide Children’s Hospital, Columbus, OH, USA; b University of Alabama at Birmingham, Birmingham, AL, USA; c Cystic Fibrosis Foundation, Bethesda, MD, USA; d University of Minnesota, MHealth Fairview Masonic Children’s Hospital, Minneapolis, MN, USA; e Harvard University, Beth Israel Deaconess Medical Center, Boston, MA, USA; f University of Pennsylvania and Children’s Hospital of Philadelphia, Philadelphia, PA, USA

**Keywords:** Elexacaftor-Tezacaftor-Ivacaftor, Cholesterol, Triglycerides, Cardiovascular disease, Metabolic disorder, PROMISE

## Abstract

**Background/Aims:**

People with CF (PwCF) have low total, high, and low density lipoprotein cholesterol (TC, HDL-C and LDL-C) and historically have had low prevalence of cardiovascular disease. More recently, cases of acute myocardial infarction are reported in PwCF. The impact of elexacaftor-tezacaftor-ivacaftor (ETI) on cholesterol and triglyceride (TG) concentrations, traditional cardiometabolic risk factors, is unknown.

**Methods/Results:**

TC, LDL-C, HDL-C, and TG concentrations were analyzed from participants enrolled in the observational PROMISE study of clinically prescribed ETI prior to and 12–18 months after initiation. Pre-ETI and follow-up concentrations were compared, and relationships between TC, LDL-C, HDL-C and TG and clinical factors were tested using linear mixed-effect models.

Fasting samples were available for 51 participants (25 M/26F, median age 17.4 y) with pancreatic exocrine insufficiency at baseline and 12–18 months after ETI initiation. TC and HDL-C were higher after 12–18 mo ETI in an unadjusted model, but with adjustment for BMI-Z, only HDL-C remained significantly higher at follow up (p < 0.05). Low HDL-C was the most common abnormality (>50 %), but prevalence of participants meeting criteria for low HDL-C did not differ between timepoints.

**Conclusions:**

In a population of youth and young adults with CF, TC and HDL-C were higher after 12–18 months of ETI, but differences in TC were attenuated with adjustment for BMI-Z. Prevalence of low HDL-C was high at both timepoints.

## Highlights

• The impact of ETI on cholesterol and triglyceride concentrations, traditional cardiometabolic risk factors, is unknown.
• Cholesterol and triglyceride levels were examined prior to and after 12–18 months of ETI.
• Total and HDL cholesterol concentrations were higher after 12–18 months of ETI as compared to baseline.
• Differences in total cholesterol seen with ETI were attenuated with adjustment for BMI-Z.

## Background

Malnutrition has been a hallmark of cystic fibrosis (CF) and is independently associated with morbidity and mortality in persons affected with this condition [1], [2], [3]. CF-related malnutrition arises from multiple factors including fat malabsorption in the setting of exocrine pancreatic insufficiency, increased energy expenditure, abnormal enterohepatic circulation of bile acids, intestinal dysbiosis, chronic inflammation, poor appetite, and cystic fibrosis related diabetes (CFRD) [2], [4]. The 2008 evidence-based clinical care guidelines recommend people with CF (PwCF) consume a high calorie and high fat diet aimed to achieve a BMI of greater than or equal to the 50th percentile of BMI in pediatrics or greater than 22 kg/m^2^ in adult females and 23 kg/m^2^ in adult males [2]. Despite dietary guidance that would be considered atherogenic in the general population, PwCF have low total and low density lipoprotein cholesterol (TC and LDL-C) [5], [6]. and have been considered protected from large vessel cardiovascular disease (CVD).

Over the past several decades, treatment advances, including the development of highly effective CFTR modulator therapies (HEMT), have led to a changing landscape in CF, with marked improvements in overall health and survival [7]. While once considered a disease of childhood, the number of adults now exceeds the number of children living with CF, and median life expectancy is 61 years [7]. Undernutrition continues to be a challenge for a subset of individuals, but its prevalence is decreasing while the prevalence of overweight and obesity are increasing [7], [8].

Improved longevity, a changing landscape of nutritional status, and co-occurrence of cystic fibrosis-related diabetes (CFRD) raise concerns for increasing CVD risk in CF. While the risk of CVD has historically been low in CF, cases of acute myocardial infarction (AMI) are now reported [9]. In a retrospective database review, Frost et al. found the prevalence of major adverse cardiac events was higher in PwCF than in a matched cohort of people without CF [10]. Older age and higher prevalence of traditional cardiometabolic risk factors including hypertension, hypercholesterolemia, and diabetes were risk factors in the cohort with CF [10].

Elexacaftor-tezacaftor-ivacaftor (ETI), considered a highly effective CFTR modulator therapy (HEMT), is associated with increases in pulmonary function, weight, and BMI [11]. Given the improved longevity in PwCF and the established relationship between cholesterol and triglyceride concentrations and CVD in the general population, understanding the relationship between use of CFTR modulator and cholesterol and triglyceride concentrations may be of relevance as longevity and overall health improve in PwCF. We conducted a secondary analysis of existing samples from PROMISE, a multi-center observational study of clinically prescribed ETI, 1) to examine changes in serum cholesterol and triglyceride concentrations at 12–18 months in adolescents and adults with CF and 2) to test the relationships of cholesterol subtypes with clinical factors traditionally associated with hypercholesterolemia and hypertriglyceridemia including age and body mass index (BMI).

## Methods

Participants were enrolled in the observational PROMISE Endocrine sub-study of clinically prescribed ETI in people with CF aged ≥ 12y and at least one F508del mutation. Methods for core assessments in PROMISE study have been previously described [11]. In this sub-study, total cholesterol (TC), high density lipoprotein cholesterol (HDL-C), low density lipoprotein cholesterol (LDL-C) and triglycerides (TG)] were assayed in fasting samples collected during oral glucose tolerance tests performed at baseline and 12–18 months at 10 endocrine sub-study centers of the 56-center study.

Serum cholesterol was measured using Roche c501 using an enzymatic colorimetric method (Indianapolis, IN). Normal cholesterol and TG concentrations were defined using age-specific, published guidelines for youth < 20 years and adults ≥ 20 years (Table 1) [12], [13].

**Table 1:** Normal cholesterol and TG levels for youth and adults as per published guidelines (TG:HDL for adults only) [12], [13].

**Category**	**Optimal Age ≥ 20**	**Optimal Age < 20**
**TC**	< 200 mg/dL	< 170 mg/dL
**LDL-C**	< 100 mg/dL	< 110 mg/dL
**HDL-C**	> 40 mg/dL	> 45 mg/dL
**TG**	< 150 mg/dL	< 90 mg/dL
**Non-HDL-C**	< 130 mg/dL	< 120 mg/dL
**TG:HDL ratio**	<3.5	**

### Statistical analyses

Analyses were performed using STATA MP/18.0. Summary statistics were used to describe clinical characteristics. Wilcoxon Sign Rank test was used to compare clinical factors between baseline and 12–18 month follow up. Data were first examined graphically for distribution, outliers, change over time and relationships with age, sex, BMI-Z and CFRD status, (BMI-Z for adults ≥ 20 years was calculated using values for 20-year-olds) and then through use of linear mixed effect models (fixed effect: visit; random effect: subject). Using these same longitudinal models, relationships of cholesterol and TG with age, FEV1 percent predicted using Global Lung Initiative Equations (FEV1pp), CFTR mutation, BMI-Z and CFRD status were tested. Covariates with significant relationships were included in final models to determine if changes in cholesterol and TG persisted over the study period after adjustment for these covariates [14]. To test whether relationships between cholesterol and triglycerides varied with these same covariates by visit, interaction terms were included in the model (e.g. i.visit##BMIZ). To compare prevalences of high TC, LDL-C and TG and low HDL-C between baseline and 12–18 month follow up, chi-squared test was used. Average TC, LDL-C, HDL-C and TG in PROMISE participants was compared to nationally representative cross-sectional data in youth without CF (NHANES) using one sample *t*-test [15].

## Results

Fasting cholesterol and TG concentrations were available for 51 participants (25 M/26F, median age 17.4 y) at baseline and 12–18 months after ETI initiation. All participants were exocrine pancreatic insufficient. CFRD was present in 14 %, and 51 % had been treated with another CFTR modulator prior to ETI initiation. Median BMI-Z increased from 0.12 to 0.54 (p < 0.01). Mean FEV_1_pp increased from 90 to 103 (p < 0.01), Table 2.

**Table 2:** Baseline demographics and clinical changes in BMI-Z, percentage of subjects who met criteria for overweight based upon BMI-Z score and FEV1pp after 12–18 months ETI. (*p < 0.05) No subjects met criteria for obesity at either time point. Overweight was defined as BMI ≥ 25 kg/m^2^ and/or BMI-Z score of ≥ 1.03, corresponding to the 85th percentile (if age < 20 years).

**Baseline Demographics (n = 51)**		
**Age**	17.4 [14.8–25.6]	
**Sex**	Male 25 (49 %) Female 26 (51 %)	
**Race**	Caucasian 49 (96 %) Black 1 (2 %), Other 1 (2 %)	
**CFRD**	No 44 (86 %), Yes 7 (13 %)	
**Prior Modulator**	No 25 (49 %), Yes 26 (51 %)	
**Second Mutation**	F508Del 24 (47 %) G551D 4 (8 %) Other minimal function 23 (45 %)	

**Clinical Changes (median [IQR])**		
	**Baseline**	**12**–**18 months**
**BMI-Z**	0.12 [-0.33–0.78]	0.54 [-0.09–1.10]*
**% Overweight**	13 %	27 %
**FEV_1_pp**	90 [69–102]	103 [93–112]*

**Fig. 1:**
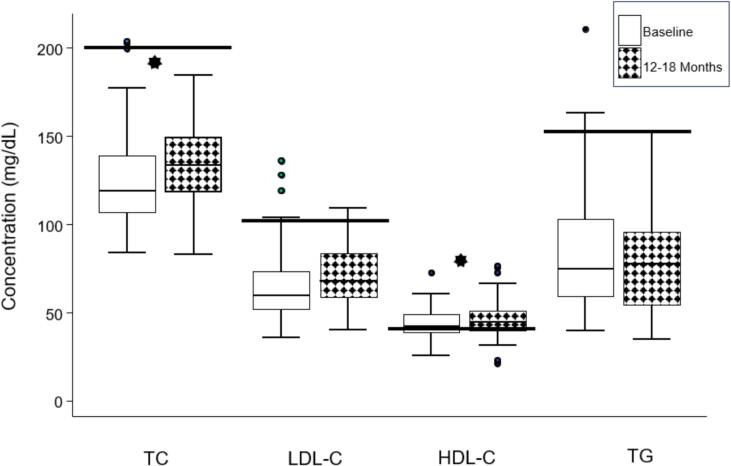
Aggregate TC, LDL-C, HDL-C and TGs at baseline and 12–18 months post-EIT initiation. Bold, horizontal lines represent ATP III upper limit of normal for general population (lower limit of normal for HDL-C).

Concentrations of TC and HDL-C were higher (p < 0.05), and LDL-C tended to be higher (p = 0.05) after 12–18 months of ETI (shown graphically in Fig. 1, and in an unadjusted, mixed-effect model in Table 3). Cholesterol and TG concentrations were not associated with age, F508Del homozygosity, sex, prior modulator use, or FEV_1_pp (data not shown).

**Table 3:** Cholesterol and TG concentrations in mg/dL at baseline and after 12–18 months of treatment with ETI, using a mixed effect, BMI-Z adjusted model. Results are expressed as mean [95 % confidence interval]. P values < 0.05 were considered significant. No significant relationships were found between cholesterol and TG concentrations and age or pulmonary function (age, sex, CFTR mutation, CFRD status and FEV1pp adjusted models tested, but not shown).

**Lipid**	**Baseline**	**12**–**18 months**	**p**
TC (Optimal < 200 mg/dL)	126 [118–133]	133 [126–140]	0.02
BMI-Z Adjusted TC	126 [118–134]	133 [126–140]	0.07
LDL-C (Optimal < 100 mg/dL)	65 [59–71]	70 [65–75]	0.05
BMI-Z Adjusted LDL-C	66 [60–72]	70 [65–75]	0.17
HDL-C (Optimal > 40 mg/dL males, > 50 mg/dL females)	43 [41–45]	46 [43–49]	0.01
BMI-Z Adjusted HDL-C	43 [41–45]	46 [43–49]	0.004
TG (Optimal < 150 mg/dL)	85 [75–95]	86 [71–100]	0.92
BMI-Z Adjusted Triglycerides	87 [77–97]	84 [70–97]	0.54
Non-HDL-C (Optimal < 130 mg/dL)	82 [75–90]	87 [81–93]	0.11
BMI-Z Adjusted Non-HDL-C	84 [76–91]	87 [81–93]	0.34
TG/HDL-C Ratio (Optimal ratio not published)	2.0 [1.8–2.4]	2.1 [1.5–2.7]	0.87
BMI-Z Adjusted TG/HDL-C Ratio	2.1 [1.8–2.5]	2.0 [1.5–2.6]	0.67

**Fig. 2:**
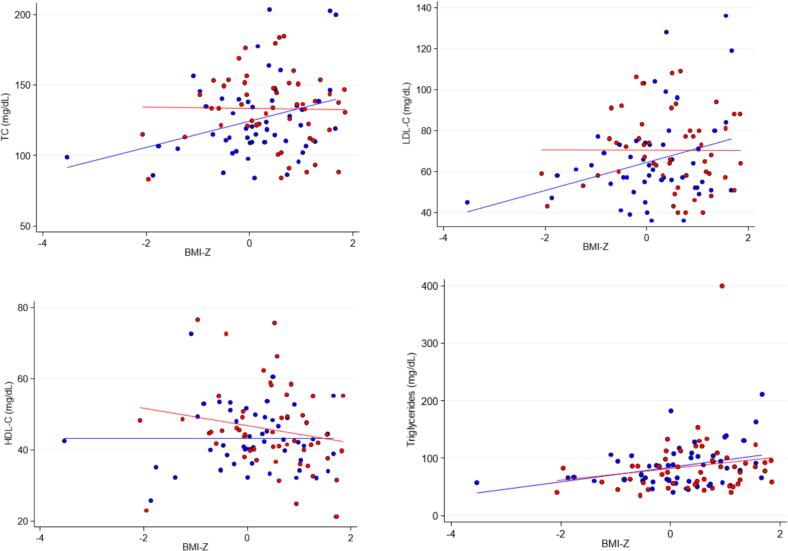
Scatter plots of cholesterol and TG by BMI-Z, with line of best fit shown by solid lines at baseline (blue) and follow up (red). The relationships of cholesterol and TG concentrations with BMI-Z did not vary with visit (p for interaction > 0.05), but consistent with graphical inspection, the interactions neared statistical significance for TC and LDL-C (both p = 0.07). Non-HDL-C and TG-HDL ratio (not shown) were not different between baseline and follow up. (For interpretation of the references to colour in this figure legend, the reader is referred to the web version of this article.)

With adjustment for BMI-Z, only HDL-C remained higher at 12–18 months (p < 0.05). TC and LDL-C tended to be higher following 12–18 months of ETI except at higher BMI-Z (p = 0.06 and 0.08 respectively, shown graphically in Fig. 2 and adjusted model in Table 3).

Cholesterol and TG concentrations did not differ by CFRD status, and, although graphical inspection suggested their increases were magnified in the seven participants with CFRD, 12–18 month post-ETI changes in cholesterol and TG did not vary by CFRD status (Supplemental Table 1, Supplemental Fig. 1).

The prevalence of abnormal cholesterol and TG concentrations, as defined by age-specific reference ranges (≥or < 20 years) for the general population, did not differ between baseline and 12–18 month post-ETI (all p > 0.05, Supplemental Table 2). Low HDL-C was the most common abnormality with 36/51 (71 %) of participants meeting criteria at baseline and 28/51 (55 %) at follow-up (p = 0.10). Using the age-specific threshold of > 90 mg/dL to define elevated TG in youth < 20 years, TG were elevated in 13/30 (43 %) at baseline vs 11/29 (38 %) at 12–18 month follow up (p = 0.67). In adults ≥ 20 years, elevated TG (>150 mg/dL) was absent at baseline and present in 1/22 at 12–18 month follow up. TC, LDL-C and non-HDL-C were not elevated in both age groups prior to and following ETI treatment (Supplemental Table 2). TC, LDL-C, HDL-C were lower than mean concentrations for a healthy population of youth (compared to NHANES reference population) [15].

## Conclusions

In a cohort of youth and young adults with pancreatic exocrine insufficient CF with varying baseline pulmonary function and nutritional status, TC and HDL-C were higher, and LDL-C tended to be higher (p = 0.05) after 12–18 months of clinically prescribed ETI. Difference in TC and LDL-C between baseline and follow up were attenuated with adjustment for BMI-Z, however higher HDL-C persisted. TG, LDL-C and HDL-C concentrations were lower than mean concentrations for healthy youth ages 12–20 [15]. Using definitions of abnormal cholesterol and triglyceride concentrations for the general population, high TC and LDL-C were absent, but low HDL-C was common and persisted, and high TG was only present in youth aged < 20 years. The present study adds to the existing literature as it examined a younger, geographically diverse population at pre-determined and consistent follow-up intervals.

The impact of ETI on cholesterol and triglyceride concentrations has been examined in single-center studies with mixed results. A single-center, retrospective analysis by Patel et al (n = 137) found higher serum total, LDL, HDL and non-HDL cholesterol after initiation of ETI as compared to prior [16]. In a prospective study by Carnovale et al, of PwCF older carrying one copy of F508Del and one minimal function mutation (n = 20), after one year of ETI, TC and LDL-C were higher than pre-ETI baseline, with a non-significant decrease in HDL-C [17]. In the general population, both magnitude and duration of LDL-C elevation are related to risk of cardiovascular events, and reduction in LDL-C confers a reduction in risk of major vascular events [18]. In the present study, TC and LDL-C were within normal range at baseline and following ETI initiation, despite weight gain. In CF, high LDL-C is uncommon, but low or normal LDL-C may be falsely reassuring in regard to risk of vascular events-- in a case series of 6 adults ages 31–68 years with CF who had acute myocardial infarction, 5/6 had low LDL-C [9]. HDL-C is responsible for reverse cholesterol transport and is independently and inversely associated with CVD risk in the general population [19]. Low HDL-C is well described in CF and has been hypothesized to arise from fat malabsorption as well as from higher circulating pro-inflammatory cytokines [6], [20], [21]. Despite findings of lower inflammatory markers detected in the lungs after ETI, prior observational studies of HDL-C in older adults with CF have reported inconsistent results [22]. Petersen *et al* also found higher HDL-C following a mean of 12.2 months of ETI treatment in adults with CFRD (N = 134, median age 33 years) while Despote *et al* did not find differences in HDL-C in adults (N = 41, mean age 40 years, 23 % with CFRD) following approximately 18 months of ETI [23], [24]. While HDL-C was higher at 12–18 months post-ETI initiation in our study, the clinical significance of this finding is limited, as the prevalence of participants meeting age- and sex-specific criteria for low HDL-C was over 50 % at both baseline and 12–18 month follow up.. A single-center, observational study by Yuzyuk et al. (n = 153) demonstrated higher HDL-C levels in a cohort of PwCF treated with CFTR modulators compared to those not treated with CFTR modulators [25]. Longer term studies are warranted to assess if HDL continues to improve with more prolonged decreased inflammatory burden. Additionally, the impact of early-in-life use of ETI on HDL concentrations remains unknown.

Hypertriglyceridemia has been reported as a common lipid abnormality in CF, possibly related to the high fat diet, as well as to elevated circulating TNF-α causing hepatic lipogenesis [26]. Elevated TGs are one of the CVD risk-enhancing factors identified by the 2018 American Heart Association cholesterol guideline, although the pathophysiologic mechanism by which elevated TG confers increased CVD risk is complex and the subject of ongoing investigation [13], [27], [28]. In our study, the prevalence of high TG was low in adults, but over one third of youth age < 20 years met criteria at baseline and follow-up. The difference in prevalence of hypertriglyceridemia between age groups was largely driven by the lower reference range for youth (< 90 mg/dL) as compared to adults (< 150 mg/dL) [12], [29]. Thus, the relevance of this elevated TG in youth for CVD is unknown. doThe low prevalence of hypertriglyceridemia identified in the adult population in the present study as compared to other studies of adults with CF may be related to the age range examined, as “adults” in this study were a maximum of 25 years of age, and hypertriglyceridemia may be associated with age. Additionally, significant changes in recommendations for diet and exercise have occurred over the past several years for PwCF, and that our more contemporary population may have had less consumption of added sugars and/or more physical activity than populations examined in years prior. Future studies should examine diet and activity level, both of which may impact triglyceride levels independently of BMI [5], [30].

The interplay between markers of metabolic health with one another and with CVD risk is not well defined in CF. In the general population, obesity is associated with higher TG and TC, but lower HDL-C [31]. In an observational study of adults with CF (mean age 25.5), Bonhoure *et al* examined 290 adults with CF and found TC, LDL-C and TC:HDL-C to be higher in the subset with overweight/obesity (BMI > 27) as compared to normal (BMI 18.5–26.9) and underweight < 18.5, but no differences in HDL-C or TG [32]. In our study population, with overall normal BMI-Z, BMI-Z was positively associated with TG concentrations and TG:HDL ratio but not with TC, LDL-C or HDL-C concentrations. The differences observed in our study population may be related to the younger median age and fewer subjects at low BMI. Interestingly, when examining changes in cholesterol concentrations between pre-ETI baseline and 12–18 month follow up, differences in TC and LDL-C were attenuated with adjustment for BMI-Z.

Insulin resistance is a component of metabolic syndrome in the general population, but associations of CFRD and obesity with abnormal cholesterol and TG concentrations have not been demonstrated. Rhodes *et al* found no difference in cholesterol and TG concentrations in individuals with CF with and without CFRD [6]. While we did not find an interaction between CFRD status and changes in cholesterol and TG concentrations between baseline and 12–18 month follow up in our study population, a tendency toward larger increases in participants with CFRD following ETI initiation was suggested. Notably, the study sample analyzed was one of convenience and not powered to detect interaction based upon CFRD status. Nonetheless, these early findings raise concern for the potential convergence of diabetes and hypercholesterolemia and hypertriglyceridemia upon vascular risk.

In the general population, risk of hypercholesterolemia and hypertriglyceridemia also increases with age. Woestenenk *et al* examined cholesterol and TG concentrations in 110 youth with CF (mean age 14.3 ± 2.4 years) and found that while concentrations traditionally remained stable across the age range in males, TG concentrations were higher with increasing age in females [21]. Nowak *et al* found that TC increases with age in CF regardless of pancreatic status [20]. Frost *et al* used two large CF patient registries (total n = 11,914) to compare rates of major cardiac events in PwCF with and without traditional CVD risk-factors and found individuals with CF who had major cardiac events were older and had a higher prevalence of hypertension, hypercholesterolemia, and diabetes than pwCF without a major cardiac event [10]. We did not find relationships between cholesterol and TG concentrations and age in our relatively young population.

Limitations of the study include sample size with limited range of age, BMI and CFRD status, primarily Caucasian population and lack of placebo. Notably, sample size is a common limitation of the published studies in this field (Petersen N = 134, Despotes N = 41, both adult only). Duration of follow up is another limiting factor. Although studies of dietary interventions on cholesterol and TG concentrations have shown changes as early as 6 months, it is possible that ETI related changes may progress more slowly over time [33]. Additionally, while high TC, LDL-C and TG and low HDL are considered surrogate markers of CVD risk in the general population, no true CVD outcome measures were available in the present study. Finally, research is needed to examine the relationships between traditional cardiometabolic risk factors and CVD risk in CF with attention to non-Hispanic black and Hispanic populations who are now recognized to have not only higher prevalence of CFRD [34] but greater risk of CVD and worse outcomes in the absence of CF.

While abnormal cholesterol and TG concentrations are reported in CF, the pattern of low TC, LDL-C and HDL-C with high TGs described in PwCF is unique and its relationship with CVD is not well-defined. Thus, future studies are needed to identify novel biomarkers which may have more sensitivity and specificity in predicting risk of cardiometabolic complications in PwCF. NMR spectroscopy has been used to identify lipid subparticles that are independently associated with CVD events within BMI cohorts and may be of value in predicting CVD risk in PwCF [35]. A better understanding of cholesterol and TG in PwCF will be important for guiding CVD-risk related screening and management in an aging CF population.

## Funding source

This work was funded by grant CFF PROMISE-OGTT18K0, PI Andrea Kelly.

## CRediT authorship contribution statement

**Rosara Bass:** Writing – original draft, Formal analysis, Data curation. **Michael Stalvey:** Resources, Project administration, Methodology, Investigation, Funding acquisition, Conceptualization. **George Solomon:** Resources, Project administration, Methodology, Investigation, Funding acquisition, Conceptualization. **Steven Rowe:** Writing – review & editing, Resources, Project administration, Methodology, Investigation, Funding acquisition, Conceptualization. **David Nichols:** Writing – review & editing, Resources, Project administration, Methodology, Investigation, Funding acquisition, Conceptualization. **Sarah Jane Schwarzenberg:** Writing – review & editing, Resources, Project administration, Methodology, Investigation, Funding acquisition, Conceptualization. **Steven Freedman:** Writing – review & editing, Resources, Project administration, Methodology, Investigation, Funding acquisition, Conceptualization. **Rachel Walega:** Writing – review & editing, Project administration, Funding acquisition. **Andrea Kelly:** Writing – review & editing, Supervision, Resources, Project administration, Methodology, Investigation, Conceptualization.

## Declaration of competing interest

The authors declare the following financial interests/personal relationships which may be considered as potential competing interests: This work was funded by grant CFF PROMISE-OGTT18K0, PI Andrea Kelly The authors declare that they have no other known competing financial interests or personal relationships that could have appeared to influence the work reported in this paper.

## Glossary

CF: Cystic Fibrosis
CFRD: Cystic Fibrosis Related Diabetes
TC: Total Cholesterol
LDL-C: Low-Density-Lipoprotein Cholesterol
HDL-C: High-Density-Lipoprotein Cholesterol
TG: Triglycerides
HEMT: Highly Effective CFTR Modulator Therapies
CVD: Cardiovascular Disease
AMI: Acute Myocardial Infarction
