# Supplementary material for: Cholesterol and triglyceride concentrations following 12–18 months of clinically prescribed elexacaftor-tezacaftor-ivacaftor—PROMISE sub-study
Source: J Clin Transl Endocrinol. 2025 Apr 2;40:100391. doi: 10.1016/j.jcte.2025.100391 (PMC12005328; doi:10.1016/j.jcte.2025.100391)
Supplement: Supplementary Data 1 [file mmc1.docx]

**Supplemental Tables and Figures:**

| **Lipid** | **Baseline (mean [95% CI])** | **12-18 months (mean [95% CI])** | **p** |
| --- | --- | --- | --- |
| TC (Optimal < 200 mg/dL) | No CFRD: 126 [117-134] | No CFRD: 131 [123-138] | 0.10 |
|  | CFRD: 127 [113-1140] | CFRD: 146 [123-169] | 0.15 |
| LDL-C (Optimal < 100 mg/dL) | No CFRD: 65 [59-72] | No CFRD: 69 [64-74] | 0.10 |
|  | CFRD: 69 [64-74] | CFRD: 72 [56-89] | 0.44 |
| HDL-C (Optimal > 40 mg/dL male,  > 50 mg/d female) | No CFRD: 43 [40-46] | No CFRD: 45 [41-48] | 0.10 |
|  | CFRD: 45 [38-51] | CFRD: 52 [42-62] | 0.10 |
| TG (Optimal < 150 mg/dL) | No CFRD: 85 [76-95] | No CFRD: 84 [68-100] | 0.80 |
|  | CFRD: 81 [65-97] | CFRD: 101 [80-122] | 0.17 |

**Supplemental Table 1:** Adjusted model incorporating age and baseline BMI-Z with interaction between visit and CFRD status.

P Values < 0.05 are considered significant.


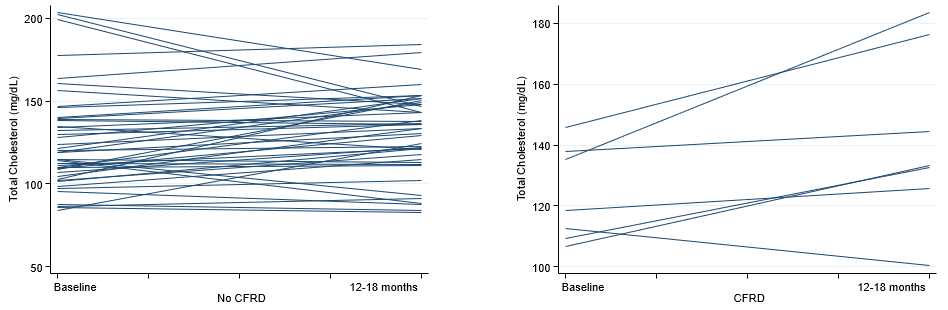

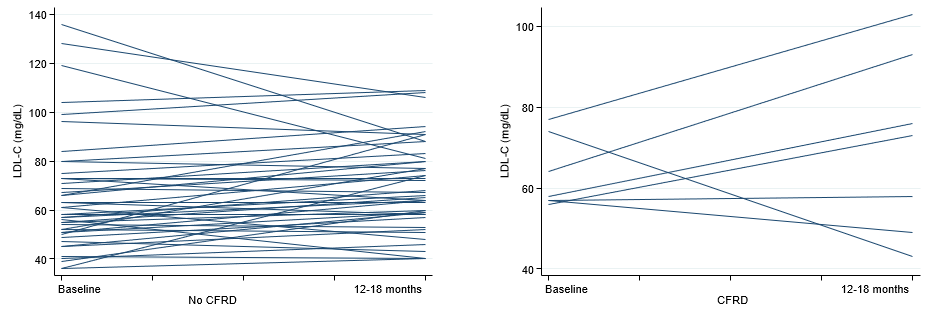

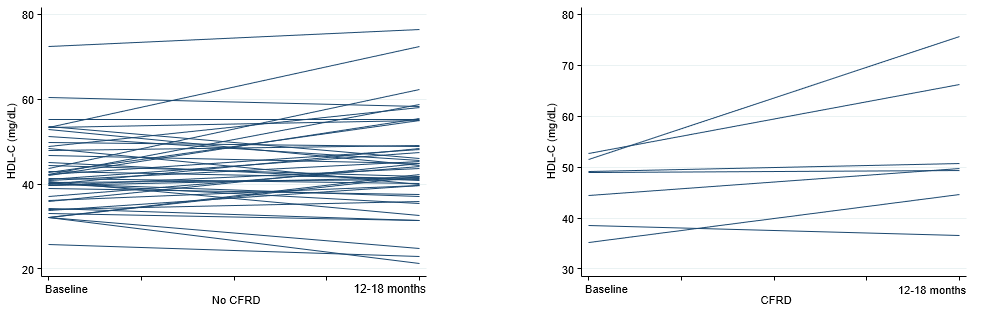

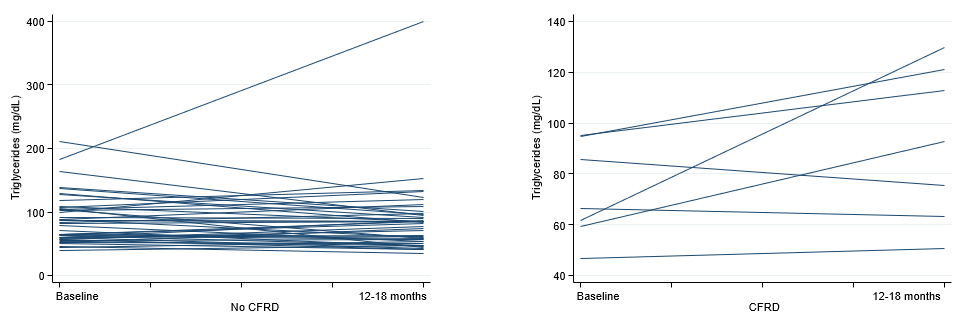


**Supplemental Figure 1:** Individual trajectories of cholesterol and TG concentrations between baseline and follow up by CFRD status.

| **Lipid** | **Reference Range for Age** | **Baseline** | **12-18 months** | **Pr** |
| --- | --- | --- | --- | --- |
| TC | Age < 20: < 170 mg/dL | 4/30 | 2/29 | 0.41 |
|  | Age ≥ 20: < 200 mg/dL | 0/21 | 0/22 | 1.00 |
|  | Total | 4/51 | 2/51 | 0.40 |
| LDL-C | Age < 20: < 110 mg/dL | 3/30 | 0/29 | 0.17 |
|  | Age ≥ 20: < 100 mg/dL | 0/21 | 3/22 | 0.08 |
|  | Total | 3/51 | 3/51 | 1.00 |
| HDL-C | Age < 20: > 45 mg/dL | 21/30 | 18/29 | 0.52 |
|  | Age ≥ 20: > 40 mg/dL (M), >50 mg/dL (F) | 4/9 M, 11/12 F | 1/9 M, 9/13 F | M=0.11  F=0.16 |
|  | Total | 36/51 | 28/51 | 0.10 |
| TG | Age < 20: < 90 mg/dL | 13/30 | 11/29 | 0.67 |
|  | Age ≥ 20: < 150 mg/dL | 0/21 | 1/22 | 0.32 |
|  | Total | 13/51 | 12/51 | 0.82 |

**Supplemental Table 2:** Prevalence of abnormal cholesterol and TG values by age at baseline and 12-18 month follow up; Pr < 0.05 considered significant). N at baseline and follow up are different as one subject turned 20 during the study.
